# Dreams share phenomenological similarities with task-unrelated thoughts and relate to variation in trait rumination and COVID-19 concern

**DOI:** 10.1038/s41598-023-33767-y

**Published:** 2023-05-02

**Authors:** Quentin Raffaelli, Eric S. Andrews, Caitlin C. Cegavske, Freya F. Abraham, Jamie O. Edgin, Jessica R. Andrews-Hanna

**Affiliations:** 1 Department of Psychology, University of Arizona, 1503 E University Blvd., Tucson, AZ 85721 USA; 2 Cognitive Science, University of Arizona, Tucson, AZ USA

**Keywords:** Psychology, Human behaviour

## Abstract

While recent neurocognitive theories have proposed links between dreams and waking life, it remains unclear what kinds of waking thoughts are most similar in their phenomenological characteristics to those of dreams. To investigate this question and examine relevance of dreams to significant personal concerns and dispositional mental health traits, we employed ecological momentary assessment and trait questionnaires across 719 young adults who completed the study during the COVID-19 pandemic, a time marked by considerable societal concern. Across the group and at the level of individual differences, dreams showed the highest correspondence with task-unrelated thoughts. Participants who self-reported greater COVID-19 concern rated their dreams as more negative and unconstructive, a relationship which was moderated by trait rumination. Furthermore, dreams perceived as more negative unconstructive and immersive in nature associated with increased trait rumination beyond variation in rumination explained by waking task-unrelated thoughts alone. Together, these results point to similarities between perceived characteristics of dreams and task-unrelated thoughts, and support a relationship between dreams, current concerns, and mental health.

## Introduction

The nature and significance of dreams have been of interest to humans since at least the heyday of Mesopotamia^1^ when theorists first recorded questions about dreams still unanswered today^2^. Despite recent scientific advances in our understanding of dreaming, it remains unclear why we dream the way we do^3^. Interesting parallels observed between the phenomenology of dreams and waking thought have shed some light on this question^4–6^, yet existing studies have been limited to relatively small sample sizes, and studies are still in their early stage of investigation into which types of waking thought are most similar to that of dreams. Beyond offering possible mechanistic insight into these questions, illuminating parallels between waking and dream cognition may also expand our understanding of factors that contribute to adaptive and maladaptive mentation.

A particular category of daytime thinking colloquially referred to as “mind-wandering”, often defined as task-unrelated thought, stimulus-independent thought, or a mix between the two^7^ (though see^8^), has been proposed to have a privileged relationship with dreaming. Fox et al.^4^ surveyed separate literatures on day and night mentation and suggested that dreams represent an extreme form of mind-wandering based on broad phenomenological similarities, shared relations with current concerns, and a common reliance on aspects of the brain’s default mode network^4,9^. Relatedly, Stickgold and Zadra^3^ proposed a direct connection between dreams and mind-wandering in their Network EXploration To Understand Possibilities (NEXTUP) model, where both phenomena are considered attempts to resolve “unfinished business” by exploring loosely connected ideas and concepts. This common emphasis on incomplete goals and unresolved concerns is also consistent with Klinger’s view that one’s repertoire of current concerns—especially emotionally salient concerns—thematically influences the content of mind-wandering and dreaming^10,11^, a theory which has received empirical support^12–18^.

Although a number of studies have noted relationships between dreams and waking cognition^19–27^, waking thoughts vary in their degree of stimulus-independence and task-unrelatedness—two independent dimensions which are operationally relevant to many views of mind-wandering^8^ (but see^28)^. Of relevance, Gross et al.^29^ compared dreams to stimulus-independent and stimulus-dependent waking thoughts, revealing that the dreams of some phenomenological characteristics were most similar to stimulus-independent thoughts, while others were most similar to stimulus-dependent thoughts. However, to our knowledge, no studies have compared dreams to waking thoughts sampled across *both* dimensions of stimulus-independence and task-unrelatedness. Existing theories lend support to a variety of hypothetical outcomes: on the one hand, task-unrelated thoughts may show a privileged relationship with dreams because of their propensity to involve topics of current emotional concern^3,10,11,30^. On the other hand, stimulus-independent waking thoughts may share the greatest similarity with dreams given a relative lack of external sensory input when sleeping. As *both* task-unrelated and stimulus-independent thoughts have been linked to the default mode network, thoughts at the intersection of these two dimensions might exhibit the strongest relationship with dreams—a hypothesis we initially endorsed. Alternatively, dream phenomenology and task-*related* thoughts may show the greatest correspondence considering that task-related thoughts represent the most common form of waking cognition^31^, and people often report dreams involving activities (such as work, school, etc.) that would be considered “tasks” when assessed in waking life^32^. Indeed, previous research on memory-related benefits of dreaming has suggested that dream content may include “unsolved” tasks, such as maze memory^33,34^. To disambiguate among these possible outcomes and address a key gap in the literature on mind-wandering and dreaming, our first study goal was to explore which categories of waking thought were most similar to participants’ typical dreams in their phenomenological qualities. We explored this question at the level of the participant group followed by an analysis of individual differences.

If dreams and mind-wandering are partially linked through their emphasis on significant current concerns, we might expect that individuals who experience more emotionally-pressing concerns would experience more emotional dreams and waking thoughts. The SARS-CoV-2 pandemic and its associated disruption to daily life marked a global concern associated with significant personal distress, particularly among young adults^35–37^. Arizona (where our study was conducted) was one of the hardest hit states by COVID-19 for its high death rates, length of school disruptions, socioeconomic disparities in impacts on health, and political discord regarding COVID-19 mitigation measures^38–40^. While many college students in Arizona were significantly affected by the COVID-19 pandemic, the degree of salient personal concern over COVID-19 also varied notably between individuals. Thus, a second goal of our study was to explore the link between emotionally-salient concerns (specifically concern over the COVID-19 pandemic) and characteristics of dreams and waking thought in a subset of participants who completed the study when COVID-related disruptions on students were highest.

Finally, our third goal was to explore the relationship between night and day mentation and a mental health trait capturing a maladaptive approach to dealing with one’s current concerns: *trait rumination*. Trait rumination, a dispositional style of thinking involving “repetitively and passively focusing on symptoms of distress and on the possible causes and consequences of these symptoms^41^,” is a transdiagnostic symptom of, and risk factor for, mood and anxiety disorders^41,42^. Although dreams have been explored in relation to maladaptive daydreaming (for an exploration of the similarity and differences with typical mind-wandering, see^43^), depression, anxiety, and other forms of mental illness^44–50^, our study extends inquiry to variation in trait rumination, and additionally asks two important questions: First, do dreams explain any *additional* variance across participants in trait rumination than waking thoughts alone? Second, does variability in trait rumination across participants moderate the relationship between COVID-19 concern and how participants perceive their dreams to be? Here, we considered that being predisposed to negative, repetitive dispositional styles of thinking may increase the likelihood that topics of current concern may manifest in one’s dreams.

To pursue these goals, we designed a three-part study to assess links between participants’ perceived daily thinking and dream phenomenology across a large sample of 719 young adults. Using ecological momentary assessment, we measured characteristics of waking thought over 1 + week with a newly-developed smartphone app called Mind Window. To assess more stable qualities of dreams while minimizing time burden to encourage a large participant sample, we also administered a self-report questionnaire to assess how participants perceive parallel characteristics of their “typical” dreams. We categorized waking thoughts into stimulus-independent task-unrelated thoughts, stimulus-independent task-related thoughts, stimulus-dependent task-unrelated thoughts, a stimulus-dependent task-related thoughts, and compared their phenomenological characteristics to those of participants’ typical dreams across the group and at the level of individual differences. In a subset of 429 participants who completed the study when COVID-related disruptions on participants were highest, we also investigated whether degree of self-reported COVID-19 concern predicted between-subject variability in the perceived characteristics of dreams, and whether this relationship was moderated by trait rumination, which we also assessed independently in relation to dreams and waking thought.

## Results

Consistent with prior lab-based studies^51^ but see^52^, stimulus-independent task-related thought was the most common type of waking cognition, characterizing a mean of 45.1% of surveys. Following in frequency were stimulus-independent task-unrelated thoughts and stimulus-dependent task-related thoughts, with a mean of about 23% each. Least common was stimulus-dependent task-unrelated thoughts, accounting for only 8.6% of surveys. Combining across categories yielded a total of 31.9% task-unrelated thoughts (compared to 68.1% task-related thoughts), and 41.7% stimulus-independent thoughts (compared to 58.3% stimulus-dependent thoughts).

### Participants view their dreams as most similar in phenomenological characteristics to their task-unrelated thoughts

**Figure 1:**
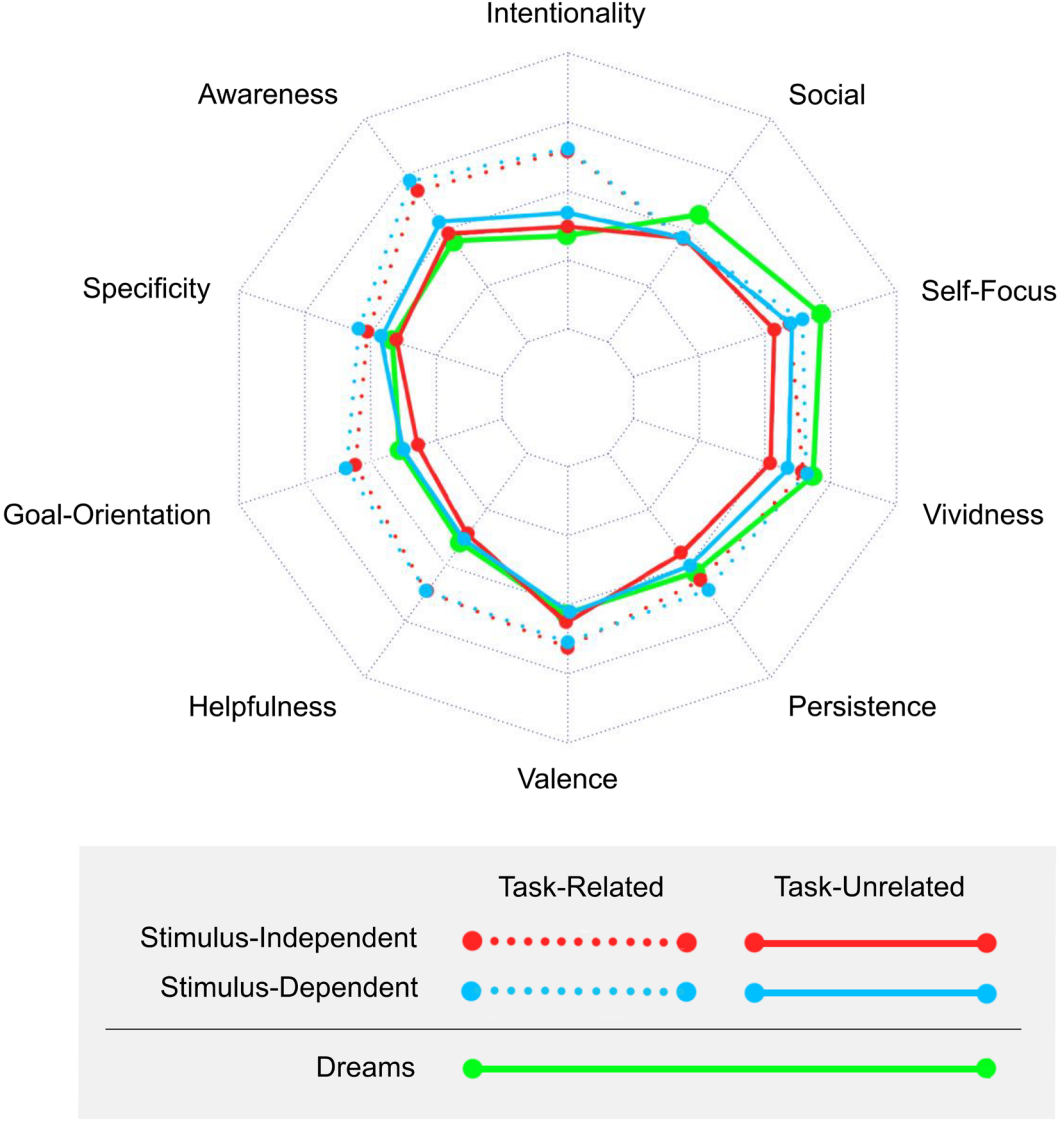
The phenomenological characteristics of dreams and different categories of waking thought. Mean ratings for dreams (green solid line) are plotted on a Spider plot, along with each of four waking thought categories. Note that the anchors for the rating scales were different for some of the characteristics (see Tables S1 and S2), but generally ranged from 0 (Not at All) to 1 (Very Much So). Valence ranged from 0 (Very Negative) to 1 (Very Positive). Stimulus dependent thoughts are plotted in red and stimulus-independent thoughts are plotted in blue. Task-related thoughts are plotted in dotted lines and task-unrelated thoughts are plotted as dashed lines. Across the phenomenological characteristics, dreams were most similar to stimulus-independent task-unrelated thought and stimulus dependent task-unrelated thought.

After characterizing each ecological momentary assessment survey as belonging to one of the 4 categories of waking thought described above, we compared each phenomenological characteristic within each waking thought category to participants’ perceived characteristics of dreaming. As can be seen in Fig. 1**,** participants viewed the characteristics of their dreams as most similar to those of task-unrelated thoughts across many of the phenomenological characteristics assessed, irrespective of the stimulus-independent or dependent nature of participants’ waking thoughts. Dreams were perceived as mildly positive in valence, moderately high on vividness, self-focus, and social content (i.e. “social-orientation”), and low-to-intermediate on their degree of intentionality, awareness, goal-orientation, helpfulness, episodic specificity and persistence.

Paired-samples Wilcoxon-rank-sum tests compared the mean values of each phenomenological characteristic for each category of waking thought to its dream-related counterpart. This analysis confirmed that task-unrelated thought categories—namely stimulus-independent task-unrelated thoughts and stimulus-dependent task-unrelated thoughts—most resembled the profile of dreams (see Table S3 and Supplementary Results for more information).

In addition to the analyses reported above, which included all participants, we repeated these analyses using different cutoffs for the minimal number of thoughts required for each participant in each waking thought category, namely 1 (n = 482), 2 (n = 327), 3 (n = 216) and 4 (n = 142), and found similar results regardless of the cutoff (see Fig. S2 and Table S3).

### Characteristics of participants’ typical dreams and task-unrelated thoughts show positive individual difference associations

Considering that waking task-unrelated thoughts showed the strongest similarities with dreams, we focused subsequent analyses on task-unrelated thoughts and examined whether the perceived characteristics of these thoughts and dreams were positively related to each other at the level of individual differences. Across individuals, we ran Spearman Rank correlations between each participant’s mean ratings across surveys for each phenomenological characteristic of task-unrelated thought in relation to their dream-related counterparts. Seven out of ten of the variables were significantly correlated across task-unrelated thought and dreams (Table 1). Six survived Bonferroni correction at a corrected alpha of *p* < 0.005: intentionality (Spearman’s *ρ* = 0.19, *p* < 0.0001), persistence (*ρ* = 0.13, *p* = 0.0007), goal-orientation (Spearman’s *ρ* = 0.14, *p* = 0.0007), helpfulness (Spearman’s *ρ* = 0.27, *p* < 0.0001), valence (Spearman’s *ρ* = 0.25, *p* < 0.0001), and social content (Spearman’s *ρ* = 0.15, *p* < 0.0001). Specificity (*ρ* = 0.07) correlated positively across dream and task-unrelated thought at the *p* < 0.05 threshold (*p* = 0.049). Notably, the same analysis performed using a cutoff of at least 5 task-unrelated thought probes for each participant yielded similar results (see Table S4).

**Table 1:** Spearman correlations between the phenomenological characteristics of task-unrelated thoughts and dreams.

Characteristic	Spearman’s ρ	*p-*value	n
Intentionality	0.19	** < 0.0001**	698
Social Orientation	0.15	**0.0001**	698
Self-Focus	0.03	0.4819	698
Vividness	0.06	0.1235	563
Persistence	0.13	**0.0007**	698
Valence	0.24	** < 0.0001**	698
Helpfulness	0.27	** < 0.0001**	564
Goal-Orientation	0.14	**0.0007**	553
Specificity	0.07	**0.0486**	698
Awareness	0.05	0.2452	556

Significant values are in bold.

Bonferroni Correction Factor = 0.005.

### Perceived dream characteristics cluster into 2 factors: positive constructive dreaming and immersive dreaming

An exploratory factor analysis was performed on the dream questions to reduce them into latent factors, which were then explored in relation to level of worry about COVID and trait rumination. The perceived dream characteristics clustered into 2 factors (Table 2). A *positive constructive dreaming* factor (Factor 1) consisted of high positive loadings for goal-orientation, helpfulness, and valence, while an *immersive dreaming* factor consisted of high positive loadings for awareness, persistence, specificity, and vividness. These 2 factors explained 19% and 17% of the variance, respectively, and the model fit for this exploratory factor analysis was within accepted standards of a good fit (*TLI* = 0.983, *RMSR* = 0.02, *RMSEA* = 0.027; see Table 2). The correlation between the 2 factors was 0.29 (*p* < 0.0001). Note that for ease of interpretation when describing relationships with rumination and COVID-19 concern, the positive constructive factor was reversed scored into a negative unconstructive dream factor by multiplying the factor score by − 1.

**Table 2:** Exploratory factor analysis on dream phenomenology revealed two latent factors.

	Factors		h2	u2	com
	1) Positive-constructive dreams	2) Immersive dreams			
Goal-orientation	0.73	0.02	0.54	0.46	1
Helpfulness	0.66	0.01	0.44	0.56	1
Valence	0.46	− 0.16	0.21	0.79	1.2
Persistence	− 0.06	0.68	0.45	0.55	1
Vividness	− 0.10	0.50	0.24	0.76	1.1
Awareness	0.24	0.47	0.33	0.67	1.5
Specificity	0.22	0.42	0.26	0.74	1.5
Variance explained	19%	17%			

Fit indices: RMSR = 0.02, Tucker—Lewis = 0.983, RMSEA = 0.027.

The phenomenological characteristics of goal-orientation, helpfulness, and valence clustered into a factor we named Positive Constructive Dreaming, which was multiplied by − 1 for subsequent analyses. The phenomenological characteristics of persistence, vividness, awareness and specificity clustered into a factor we named Immersive Dreaming. The fit indices were clearly within accepted standards of a good fit.

### Feelings of hopelessness about a global current concern, the COVID-19 pandemic, associates with negative unconstructive dreams and task-unrelated thoughts

We quantified the associations between negative unconstructive and immersive dreaming factor scores and the degree of concern participants expressed about the COVID-19 pandemic within the subset of the sample for which data was collected when COVID-related disruptions were high (n = 429). The negative unconstructive dream factor was significantly associated with participants’ level of concern about the pandemic (*r*(427 = 0.19, *p* < 0.0001), but the immersive factor did not show a significant relationship (*r*(427) = − 0.028, *p* = 0.52). Exploratory post-hoc analyses examining relationships for each phenomenological characteristic separately are reported in Table S5. As revealed by a stepwise linear regression analysis, the negative unconstructive dream factor explained a marginal degree of additional variance in self-reported level of concern about the pandemic beyond its task-unrelated thought counterparts alone (Model for task-unrelated thoughts alone: *adjusted r*^2^ = 2.8%, *p* = 0.011; Model including the addition of negative unconstructive dreams: *adjusted r*^2^ change = 0.9%, *p* = 0.059).

### Trait rumination associates with both negative unconstructive dreams and task-unrelated thoughts

**Figure 2:**
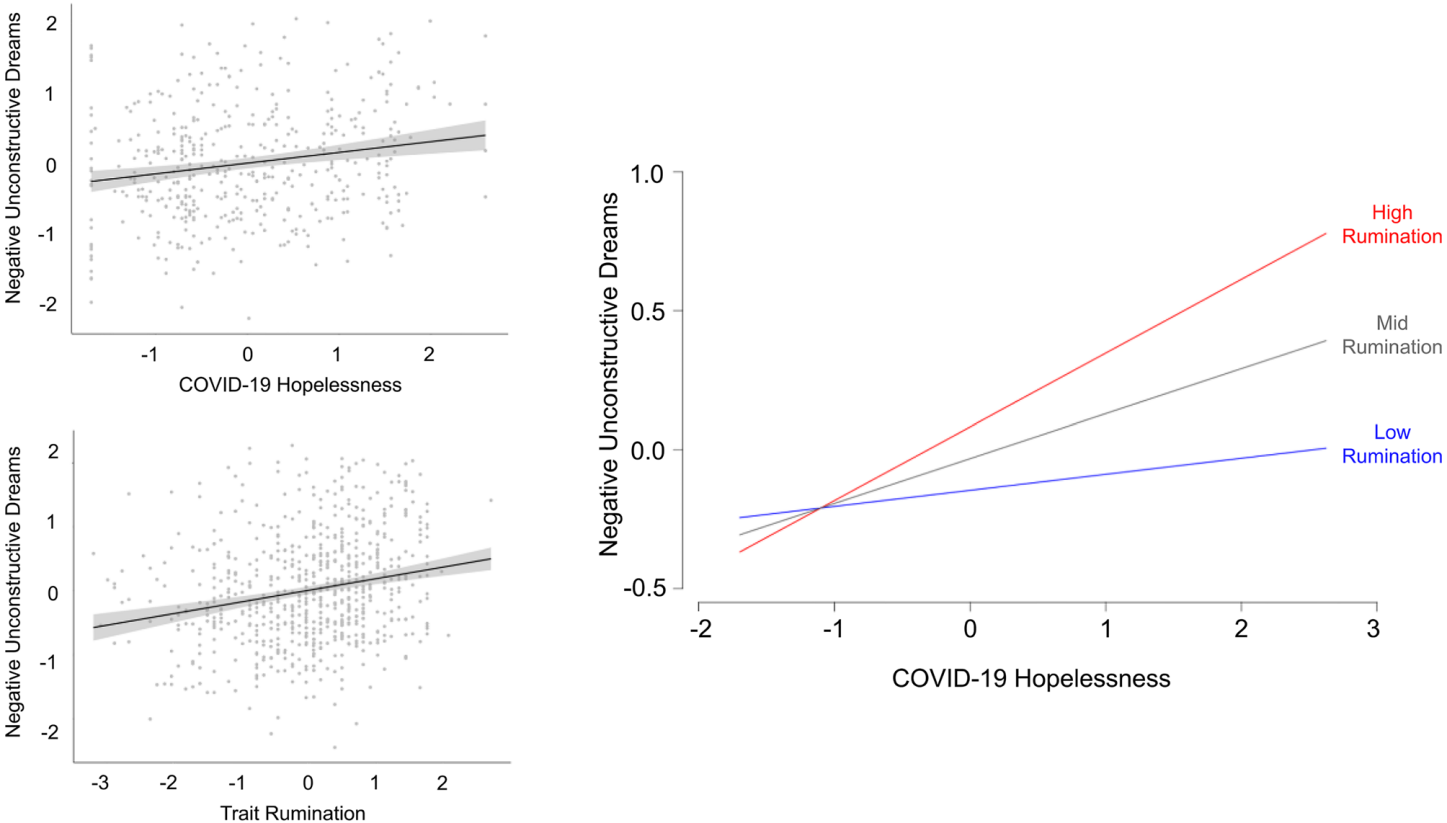
Relationship between COVID-19 hopelessness, trait rumination, and negative unconstructive dreams. There were significant positive correlations between negative unconstructive dreams and both COVID-19 hopelessness (top left) and trait rumination (bottom left) (all p’s < 0.0001). An interaction model revealed that the association between COVID-19 hopelessness and negative unconstructive dreams was exacerbated by higher level of trait rumination (right).

We next assessed whether trait rumination relates to participants’ perceived dream characteristics. Both the immersive dream factor (*r*(717 = 0.083, *p* = 0.026) and the negative unconstructive dream factor positively related to trait rumination (*r*(717) = 0.222, *p* < 0.0001, Fig. 2). Exploratory post-hoc analyses examining relationships for each phenomenological characteristic separately are reported in Table S6. Combined in the same linear model (*F*(2, 695) = 26.89, *p* < 0001), both dream factors contributed independent variance (negative unconstructive dreaming: *b* = 0.25, *t*(696) = 6.89, *p* < 0.0001; immersive dreaming: *b* = 0.16, *t*(696) = 4.32, *p* < 0.0001) and accounted for 6.92% of the variance (*adjusted r*^2^) in trait rumination. Furthermore, a stepwise regression analysis revealed that the dream factors explained variance above and beyond their task-unrelated thoughts counterpart (*adjusted r*^2^ change = 4.15%, *p* < 0.0001). The model predicting trait rumination based on task-unrelated thoughts was also significant (*adjusted r*^2^ 10.7%, *p* < 0.0001).

### Trait rumination exacerbates the relationship between COVID-19 concern and perceived dream characteristics

Finally, we examined whether having a maladaptive way of processing current concerns would exacerbate the relationship between degree of one’s worry about the COVID pandemic and negative unconstructive dreams. A test of interactions within the linear model predicting negative unconstructive dreaming from levels of trait rumination and worry about the COVID-19 pandemic revealed a significant interaction between the two predictors (*b* = 0.10, *t*(427) = 2.91, *p* = 0.004), on top of two main effects (negative unconstructive dreaming: *b* = 0.11, *t*(427) = 2.94, *p* = 0.0035; immersive dreaming: *b* = 0.16, *t*(427) = 4.06, *p* < 0.0001). This model (*F*(3, 425) = 12.74, *p* < 0001) explained 7.60% of the variance (*adjusted r*^2^). A propensity to ruminate exacerbated the negative unconstructive dreams that were associated with COVID-19 related worry.

### Controlling for individual differences in personality did not substantially affect the results

A potential limitation of our trait dream questionnaire is that participants—especially those with low dream recall frequency—may rely on their own high-level views of themselves and their thoughts (i.e. self concept or personality) to infer answers to questions about their dreams^53^. We therefore assessed participants’ Big 5 personality traits using the Ten-Item Personality Inventory^54^. We then examined relationships between personality factors and dream characteristics, and repeated previous analyses while controlling for personality trait scores.

More specifically, we first tested the existence of a relationship between each of the dream factors revealed by the exploratory factor analysis and each of the Big 5 personality scores from the Ten-Item Personality Inventory. While negative unconstructive dreaming was significantly related to all of the personality scores but agreeableness (all *p*’s < 0.004), immersive dreaming was only significantly correlated with openness to experience (*r* = 0.112, *p* = 0.001) (see Table S7). To control for the potential confounding effects of personality on how participants answered the dream questionnaire, we repeated some of the analyses while accounting for participants’ scores on the Ten-Item Personality Inventory. We ran multiple linear regression models predicting each of the task-unrelated thought characteristics variables from their dream-related counterparts while including the five personality trait scores as covariates (see Table S8), replicating replicated results from Table 1. All dream variables that were significantly correlated with their task-unrelated counterparts remained significant predictors after accounting for the 5 personality scores, while the dream variables that were not significantly correlated with their task-unrelated counterparts remained not significant predictors after accounting for personality.

Similarly, trait rumination remained significantly related to both negative unconstructive and immersive dreams (both *p*’s < 0.001), consistent with what we observed prior to controlling for personality. Finally, once controlling for all 5 personality scores, negative unconstructive dreams became only marginally related to levels of worry/concern about COVID-19 (*p* = 0.055) while immersiveness remained non-significantly related to COVID-19 concern (see Table S8). However, when only controlling for neuroticism (the personality trait most strongly related to negative unconstructive dreams), the relationship between COVID-19 concern and negative unconstructive dreams remained significantly associated (*p* = 0.0071).

## Discussion

Across a large, diverse cohort of young adults, we employed ecological momentary assessment and trait questionnaires to measure perceived dream characteristics in relation to four categories of waking thoughts, self-reported concern over the COVID-19 pandemic, and individual differences in trait rumination. A number of novel findings emerged: (1) At the group level, task-unrelated thoughts (regardless of their stimulus-dependence) showed the strongest perceived similarities to dreams. (2) At the individual level, the way participants viewed many phenomenological characteristics of their typical dreams positively associated with similar characteristics of task-unrelated thoughts. (3) Participants who expressed more concern over the COVID-19 pandemic experienced more negative unconstructive dreams. (4) Participants with higher trait rumination rated their dreams as more negative, unconstructive and immersive, and dreams explained additional variance in trait rumination than waking task-unrelated alone. (5) Trait rumination moderated the relationship between COVID-19 concern and perceived dream characteristics, such that more ruminative individuals displayed a stronger positive relationship between COVID-19 concern and negative unconstructive dreams. Though correlational and not directly assessing the mechanisms through which these cognitive states arise, these results provide converging support for theories suggesting that dreams and mind-wandering share common mechanisms and a common focus on significant current concerns^3,4,6,11^.

### Participants perceived their dreams as most similar to the phenomenological characteristics of task-unrelated thoughts

Our results join a body of work reporting associations between day and night cognition^19–27^. Here we aimed to extend beyond this existing literature by comparing perceived dream characteristics to dimensions of waking thought studied extensively in the literature on mind-wandering—task-unrelatedness and stimulus-independence—as well as by examining dispositional and concern-related predictors of perceived dream characteristics across individuals. Participants’ perceived characteristics of their dreams most closely resembled those of task-unrelated waking thoughts, regardless of whether such thoughts were directed towards internal representations or external sensations. Dreams and task-unrelated thoughts also showed significant associations at the level of individual differences for most phenomenological characteristics sampled—a finding that strengthened when controlling for variance in ratings across daily surveys.

Collectively, these findings are consistent with theories that dreaming may be mechanistically related to mind-wandering^4,5^, yet the nature of such mechanisms linking the two forms of mentation are still unclear and should be explored in future work. Stickgold and Zadra’s NEXTUP theory^3^ and Klinger’s theory of dreams^11^ suggests that both task-unrelated thoughts and dreams share loose associative thinking processes that may be evoked to help process current concerns. To provide further mechanistic insight into the similarities between dreams and waking thought, future studies could more directly investigate dynamic and associative processes of these two forms of mentation. While many task-unrelated thoughts emerge spontaneously and are likely to transition dynamically and with ease, other task-unrelated thoughts may be guided by deliberate or automatic constraints that restrict how thoughts arise and unfold over time^8,55^. Recent neurocognitive models suggest that dreams may be more closely related to spontaneous waking thoughts than deliberately or automatically-guided thoughts^8,56^, a prediction supported by relatively low phenomenological ratings of dreams on intentionality and goal-orientation, and neutral on valence.

Both dreams and task-unrelated thoughts were rated as low to intermediate on their degree of intentionality, awareness, goal-orientation, helpfulness and episodic specificity. Additionally, both dreams and task-unrelated thoughts were rated neutral to slightly positive in valence across participants, similar to prior independent work on dreams and self-generated cognition^29,57,58^. The relatively low level of dream intentionality and awareness is consistent with activity reductions of the prefrontal cortex during REM sleep in non-lucid dreamers^4^ and with observations that a significant fraction of task-unrelated thoughts are dream-like^59–61^ and lack meta-awareness^62^. Given the general nature of our dream questionnaire, the neutral rating may reflect an average of multiple positive and negative emotional states, in line with some studies emphasizing a stronger degree of positive and/or negative emotions in dreams (see^4,63^). Diverging from task-unrelated thoughts, however, dreams were rated moderate to high on vividness, self-focus, and social-orientation, consistent with their proposed personal and social significance^64–68^, as well as with a heightened involvement of the default mode network and high level visual areas during REM sleep^4,5,56^. Interestingly, the high level of dream vividness was on par with that of both types of task-related rather than task-unrelated thoughts.

As a whole, these results extend prior work comparing the phenomenology waking thought and participants’ typical dreams^19–27^ by considering different forms of the former and by providing a more granular picture of the phenomenology of the latter. While our findings of similarities between dreams and task-unrelated thoughts are in line with theories that emphasize the role of mind-wandering in dreams^4,5,11^, it was surprising to us that for many characteristics, stimulus-independent task-unrelated thoughts (as compared to stimulus-dependent task-unrelated thoughts) did not demonstrate a privileged status in their similarity to dreams, especially considering that the external sensory input that characterizes stimulus-dependent task-unrelated thoughts (a.k.a. “external distractions”) is typically absent during sleep. These findings deserve investigation in future studies considering that stimulus-dependent task-unrelated thoughts was the least frequently experienced category (8.6% of surveys answered) and results may not generalize as well across different contexts. Alternatively, participants may exhibit diffuse attention to both external and internal representations, a possibility for which our method of partitioning a continuous scale into categorical groupings overlooks^69^. Towards this end, future work could explore the full range of each dimension of task-relatedness and stimulus-dependence.

### Adults with higher trait rumination experience their dreams as more negative, unconstructive, and immersive

Prior work has shown that the qualities of dreams relate to person-level traits, including traits relevant for mental health^70^. For example, individuals with depression tend to have negative or less contextually detailed dreams^71–73^, whereas individuals experiencing the manic phase within their bipolar disorder report bizarre and improbable dreams^73^. Similarly, individuals with anorexia often experience dreams involving a distorted body perception^74^, and changes in mental health status over time often associate with concordant changes in dream experiences^73,75–77^. Here, we extended empirical investigation of dream phenomenology in two ways. First, our results extended to a transdiagnostic symptom and risk factor for mental illness: *trait rumination*. Trait rumination is considered a dispositional style of thinking in which people dwell on their personal problems and concerns in a manner that disrupts focus on the task at hand^78^, exacerbates distress, and prolongs symptoms of depressive episodes^79^. In contrast to more constructive forms of repetitive thought, trait rumination is considered a maladaptive way of attempting to solve current concerns^80^. Here we observed that increased trait rumination was associated with dreams being rated as more negative, unconstructive, and immersive. While we focused on rumination given that ruminative thoughts often involve dwelling on one’s past and current concerns^41,58,81^, future studies could examine whether dreams can differentiate between different forms of negative repetitive thinking, such as between rumination and worry. Second, our results suggest that there is a congruence between characteristics of task-unrelated thoughts and dreams in typical cognition. There was indeed an association between both forms of cognition for most of the characteristics we measured. Future studies could examine whether individual differences in these features of task-unrelated thought predict processing of similar characteristics of corresponding external stimuli. For instance, Ho and colleagues^82^ showed that individuals with a tendency towards more off-task social content showed a stronger neural response to faces rather than other real world stimuli.

Although the link between dream characteristics and trait rumination and task-unrelated thought are central findings of our study, positive relationships could be driven by inaccuracies in our unvalidated and exploratory dream sampling approach in which we asked participants to reflect on the general nature of their dreams before using the Mind Window app. If participants do not have accurate insight into their typical dreams, they might answer the dream questionnaires in accordance with how they view themselves and their personality. Importantly, significant relationships between dream characteristics and trait rumination and task-unrelated thought ratings persisted when controlling for all five personality factors (see Table S8). These findings lend validity to our general dream questionnaire and suggest that the relationships we observe represent the amalgamation of participants’ recalled dreams (and not simply how one views one’s self or one’s thoughts independent of the nature of their dreams). This is important because our general dream questionnaire was critical to our success in recruiting a large diverse cohort of participants and to capturing more dispositional styles of dreaming that may be difficult to capture with methods assessing both dreams and daily thoughts on a day-to-day basis.

### Heightened concern over the COVID-19 pandemic was associated with more negative, unconstructive dreams

Although we did not sample participants’ full repertoire of current concerns, a subset of 429 of our study participants completed the study when disruptions to the daily life of undergraduates from the COVID-19 pandemic were highest. As a measure of variation in personal emotional concern over the pandemic, we further assessed perceived hopelessness in relation to the pandemic’s impact on one’s lives. Interestingly, participants who reported more concern over the near future of COVID-19 also reported more negative, unconstructive styles of dreaming. Indeed, the COVID-19 pandemic has been associated with an increased frequency of negatively-valenced dreams, nightmares^83–85^, and COVID-19 related dream content^86–89^. Furthermore, studies pre-dating the pandemic have shown that transient life stressors can affect dreams^16–18^, and some studies have directly linked dreams to individuals’ most pressing concerns^13,14^. Together, these empirical efforts converge towards the idea that current concerns and dreams bear a non-random relationship lending credence to the theory of dreaming outlined above^3,4,11^. Providing further support for similarities between dreams and task-unrelated thoughts, the phenomenology of task-unrelated thoughts also related to levels of COVID-19 concern and trait rumination. Notably, dreams contributed common but also a small but significant amount of unique variance in relation to trait rumination (and marginally significant with regards to COVID-19 related worry) when compared to daily thinking alone, in line with suggestions that dreams represent a particularly special means to process difficult emotional experiences^90,91^.

Interestingly, the relationship between COVID-19 concern and negative unconstructive dreams was moderated by trait rumination, such that as trait rumination increased, participants demonstrated stronger positive relationships between pandemic worry and negative unconstructive dreams. Indeed, people with pre-existing mental health conditions were impacted more negatively by the COVID-19 pandemic^92–94^, joining a body of work suggesting that dispositional mental health risk factors (including trait rumination) may lower one’s resilience to the occurrence of challenging life events^95,96^. Although far from illuminating any causal relationships, our findings hint at the relevance of dreams to this topic, as the act of repetitively dwelling on one’s problems and concerns may increase the likelihood that topics of concern will manifest in one’s dreams. Indeed, in one empirical study, individuals assigned to ruminate about an intrusive thought before sleep were more likely to experience threatening and negatively-valenced dreams^97^. Longitudinal analysis of the reciprocal relationship between ruminative thoughts and ruminative dreams would be an interesting direction to unpack in future research. Additionally, our results complement prior work demonstrating that the COVID-19 pandemic brought changes in daily activities and everyday thought patterns^98^, as well as the content and quality of dreams^83–89^. As we did not collect systematic data on what participants were doing when they answered the momentary surveys, future work could explore whether important changes in daily life routine impact task-unrelated thoughts and dreams.

### Limitations and future directions

We acknowledge some important limitations in our current design. First, we acknowledge that some of the dream characteristics may be difficult for participants to report on in the manner assessed here. Skeptical readers may want to focus their attention on more commonly studied dream characteristics such as valence, which was related to COVID-19 concerns and rumination as hypothesized and had the second highest correlation with its task-unrelated thought counterpart (see Table S5 and Table S6). Despite this putative rating difficulty, it is encouraging that the characteristics of dreaming clustered meaningfully as positive constructive (i.e. positive valence and helpful) and immersive factors (i.e. vivid and contextually specific).

Second, the method we used to assess the characteristics of waking thoughts differed from our assessment of dream phenomenology. Although we administered a parallel set of self-report questions addressing phenomenological variables across dreams and waking thoughts, we assessed waking thoughts longitudinally in daily life via ecological momentary assessment, whereas we administered a single questionnaire to assess the phenomenology of participants’ “typical” dreams at the beginning of the study. Importantly, the objective of our study was not to examine daytime sources of night-to-night variation in dream content (as predicted by the continuity hypothesis of dreams^99–101^), but rather to assess more stable properties of waking and dreaming mentation, while minimizing the time burden on participants to facilitate collection of large sample sizes. Considering our goal to break down waking thoughts into 4 distinct classes of cognition, adopting the same methodology across day and night mentation would have required either administering 4 separate trait-level waking thought questionnaires (one pertaining to each class of cognition under consideration), or following waking thoughts and dreams across multiple days and nights to extract more stable characteristics relevant for individual differences. Unfortunately, the former approach would be difficult for participants to distinguish between different classes of waking thoughts, whereas the latter approach would add a heavy participant burden on top of an already time-intensive study. Nightly awakenings to assess dreams—considered the most accurate assessment approach for dream content—also comes with additional limitations, including fragmented sleep that may affect cognition, mood, and thoughts during the subsequent day^102^. As mentioned above, nightly awakenings are also very time demanding, including in their need for polysomnographic monitoring, and they are sensitive to transient factors^103^ that are beyond the scope of the present study. Similarly, the use of morning retrospective dream diaries require a large numbers of dreams over time to establish reliable trends^104,105^ as smaller dream samples have poor restest validity due to high levels of fluctuation in dream content^104–107^. This lengthy data collection negatively affects the motivation of participants as evidenced with important drop in the number and length of dream recall reported between the first and second week of data collection^105^. Considering the drawbacks of these approaches, while also balancing our desire for larger more representative participant samples, we opted to use a general trait questionnaire to sample dreams and an ecological momentary assessment procedure to sample everyday thoughts. Nevertheless, our use of diverging methods may reduce our power to find relationships between dreams and waking thought, a consequence that should be noted when interpreting our fairly small study effect sizes.

One important limitation of using a general method of assessing dreams is that it may be sensitive to the most salient and memorable features of participants’ dreams, possibly underestimating their more mundane characteristics. Additionally, participants may have partially based their answers to the dream questionnaire on their impressions of the dream-like qualities of their waking thoughts. Nevertheless, there are reasons to believe that participants may be reasonably able to assess the quality of their dreams using trait questionnaires. Prior studies have shown significant correlations when assessing characteristics of dreams with trait questionnaires and morning dream diaries within the same participants, especially in individuals who do not have poor dream recall^106,108,109^. Additionally, a number of studies have shown predictive validities in the use of trait dream questionnaires to predict individual differences in diverse outcomes such as aphantasia, olfactory experiences, and bodily self-consciouness^110–112^. Despite the limitations noted above, sampling dream content using a nightly awakening procedure would yield more accurate insights into the content of the dream itself, and would also make it feasible to compare dream content across different stages of sleep, an important feature of some theories of dreaming such as the NEXTUP theory^3^. Future studies could adopt a longitudinal dream sampling approach to compare the dreams of REM and non-REM sleep in relation to task-unrelated and task-related waking thoughts. Our results are correlational and indirect, as we did not directly assess dream content or the full repertoire of participants’ current concerns. Nevertheless, our results establish important initial empirical evidence regarding the relevance of task-unrelated thoughts and ruminative thinking to the perceived characteristics of dreams.

## Materials and methods

### Participants

836 participants from the University of Arizona’s Psychology undergraduate student pool participated in this experiment in exchange for course credits. 719 of these participants completed at least 10 ecological momentary assessment surveys and were retained for subsequent analysis (see below for details). The mean age of this analyzed participant cohort was 19.82 years (SD = 4.13, 550 females, 152 males, 16 nonbinary, transgender, or self-described). Despite the relatively restricted age range (note, only 22 participants were over the age of 30), participants were racially/ethnically diverse, with only 51% of participants identifying as Non-Hispanic White (for a racial/ethnic breakdown, see Fig. S1**)**. Participants were required to be fluent in English and at least 18 years of age. Data collection began in August 2020. The analyses in this study used a download of the data from May 09, 2022. Written informed consent was obtained from all participants and all procedures were performed in accordance with the relevant guidelines and regulations and approved by the University of Arizona’s Institutional Review Board.

### Momentary waking thoughts

Daily thoughts were collected via the ecological momentary assessment smartphone app, Mind Window, developed by authors ESA and JAH. Participants downloaded the app (free of cost) to their iOS or Android smartphones, completed a battery of sociodemographic and trait questionnaires within the app (most not part of this study) and specified the time they typically wake up and go to sleep. Mind Window then sent participants six notifications (i.e. surveys) per day, quasi-randomly dispersed within equal intervals during participants’ waking day. The notification disappeared 10 min after it was sent and participants were asked to respond to the survey immediately after it arrived to their phone. The ecological momentary assessment surveys prompted participants to answer a variety of questions about their thoughts and feelings in the moments “just before the notification.” Each check-in consisted of 14, randomly ordered, questions—twelve ‘core’ questions that were common across all check-ins and two additional questions randomly selected from eight rotating questions (see Table S1 for the full list of survey questions). Due to their theoretical and statistical similarities, spatial and temporal specificity were averaged into a single episodic specificity variable.

“Two core survey questions inquired about task-relatedness and level of perceptual coupling. Despite our assessment of these metrics on a continuum, for ease of interpretability in consideration of our study goals, we decided to dichotomize and cross the two dimensions by splitting the scale in half. This yielded four independent classes of cognition based on prior literature^51^: stimulus-independent task-unrelated thoughts, stimulus-dependent task-unrelated thoughts, stimulus-independent task-related thoughts, and stimulus-dependent task-related thoughts (e.g. a thought was categorized as stimulus-independent task-unrelated thought if its level of perceptual coupling was less than 0.50 and its level of task-relatedness was less than 0.50). Participants were asked to complete as many check-ins as possible over 7 days, though many participants continued to use the app. Additional surveys completed by participants were included in the present study for up to 14 days following the beginning of the experiment. In order to facilitate stable estimates of everyday thought, participants who answered fewer than 10 surveys were removed from the dataset, leaving 719 participants out of the initial 836 with a mean of 31.32 surveys (SD = 10.80). Although this compliance seems low in many people, it is important to note that because of the multi-faceted nature of our study (extending beyond that of dreams and waking thought), we encouraged participants to participate in the study even if they did not intend to complete the ecological momentary assessment portion of the experiment. Participants were awarded credit in accordance with the portions of the study they completed.

### Self-reported dream characteristics

In a separate survey completed at the onset of the study, participants were asked to reference their typical dreams to a similar set of phenomenological variables as those asked in reference to participants’ momentary thoughts (see Table S2 for the questionnaire), including: (1) *intentionality*, (2) *awareness*, (3) *persistence*, (4) *spatial specificity*, (5) *temporal specificity*, (6) *vividness*, (7) *goal-orientation*, (8) *helpfulness*, (9) *valence*, (10) *self-focus*, and (11) *social orientation*. This allowed us to examine convergence or divergence between dreams and waking thoughts across a broad range of qualities, extending beyond characteristics studied most frequently in the dream literature (e.g. valence). We opted for a trait-like dream questionnaire separated in time from the ecological momentary assessment study because we aimed to (1) minimize study time demands as much as possible to encourage a large sample size, and (2) assess stable properties of dreams without influencing waking thoughts. In contrast, a method employing nightly awakenings to assess dream content would have substantially reduced our sample size, and may have affected participants’ waking thoughts by disrupting sleep or providing a possible scaffold to answer the daily thought probes.

### Trait rumination

The 12-item trait rumination subscale, from the Rumination-Reflection Questionnaire^113^ (RRQ), was administered via Qualtrics to assess participants’ tendency to ruminate, or “dwell” on past experiences or unwanted thoughts. Questions were answered on a 7-point Likert scale from strongly disagree to strongly agree. The reliability of the questionnaire in our sample was excellent (ICC = 0.909, CI_95_ = [0.898, 0.919]).

### Individual differences in COVID-related concern

A subset of our data (n = 429, M age = 19.56, SD age = 4.21, 317 females, 104 males, 8 gender nonbinary or other) was collected during COVID-related disruptions of daily life and included an extra Mind Window questionnaire pertaining to participants’ experience of the impact of COVID on their life. One question assessed individual differences in perceived level of concern about the COVID-19 pandemic, which we felt was important to assess in relation to dream-related phenomenology: “Looking ahead to the next few weeks, how HOPEFUL do you feel?”. The question was asked in reference to the COVID-19 pandemic and its impact on participants’ lives. Therefore, lower values on this question likely reflect how emotionally concerned participants feel about the pandemic at the time participants completed the survey. The item was rated on a sliding scale with 5 anchors (not at all, minimally, somewhat, quite a bit, extremely) and was converted to a number from 0 to 1. For ease of interpretation in subsequent analysis, this item was reverse scored such that higher scores reflect more worries about the future of the pandemic. The average level of COVID-19 concern was 0.39 (SD = 0.24).

### Ten-Item Personality questionnaire

The Ten-Item Personality Inventory^54^ was included to address the concern that participants may have difficulties self-reflecting on their “typical” dreams, and therefore may answer the questionnaire by referencing their personality traits. Including a measure of personality allowed us to examine whether relationships between characteristics of dreams and waking thought, or other measures of interest, persist when controlling for individual differences in personality. The Ten-Item Personality Inventory yields scores for neuroticism, extraversion, agreeableness, conscientiousness and openness to experience, has been reported to have good validity^114^ and been successfully translated in other languages^115,116^.

### Statistical analysis

Due to the non-normal distributions of the dream ratings, statistical comparisons between dreams and the 4 categories of waking thoughts were assessed via Wilcoxon-Rank-Sum paired tests. To account for 40 statistical tests, we also applied Bonferroni correction, yielding a corrected alpha of *p* < 0.001. For the same reasons, most of the correlations involving the dream variables were Spearman correlations, and Bonferroni correction for 10 tests was applied in the analysis investigating the strength of the associations between characteristics of task-unrelated thoughts and participants’ typical dreams (corrected alpha of *p* < 0.005).

To examine interrelationships between participants’ perceived dream characteristics and to minimize the number of statistical tests performed in relation to trait-level factors beyond dreams, an exploratory factor analysis was conducted on questions in the dream survey (see Supplementary Information for more details). To determine whether the dream factors explained variance in these variables above and beyond that of waking thoughts, we ran a series of stepwise regression models. First, a baseline model predicting either level of worry about COVID or trait rumination with the variables that made up the individual dream factor was compared to a model on top of which the dream factor was added. Finally, as we hypothesized that a maladaptive approach to dealing with concerns would exacerbate the effect of a specific current concern like worry about COVID-19 on dream phenomenology, we conducted an interaction model predicting each dream factor by trait rumination and level of worry about COVID-19. To prevent multicollinearity issues with the interaction model, all variables were z-scored.

## Supplementary Information

Supplementary Information.

## Data Availability

The data that support the findings of this study are available from the corresponding author upon reasonable request.
