# Supplementary material for: Dreams share phenomenological similarities with task-unrelated thoughts and relate to variation in trait rumination and COVID-19 concern
Source: Sci Rep. 2023 May 2;13:7102. doi: 10.1038/s41598-023-33767-y (PMC10152021; doi:10.1038/s41598-023-33767-y)
Supplement: Supplementary file 1 — Supplementary Information. [file 41598_2023_33767_MOESM1_ESM.docx]

**Supplementary Information for**

Dreams share phenomenological similarities with task-unrelated thoughts and relate to variation in trait rumination and COVID-19 concern

Quentin Raffaelli*^1,2†^, Eric S. Andrews^1,2†^, Caitlin C. Cegavske^1^, Freya F. Abraham^1^, Jamie O. Edgin^1^ & Jessica R. Andrews-Hanna*^1,2^

^†^ Equal author contributions

^1^ Department of Psychology, University of Arizona, Tucson, AZ, USA

^2^ Cognitive Science, University of Arizona, Tucson, AZ, USA

**Contact:**

Quentin Raffaelli: [quentinraffaelli@arizona.edu](mailto:quentinraffaelli@arizona.edu)

University of Arizona, Department of Psychology, 1503 E University Blvd., Tucson, AZ 85721

Jessica R. Andrews-Hanna: [jandrewshanna@arizona.edu](mailto:jandrewshanna@arizona.edu)

University of Arizona, Department of Psychology, 1503 E University Blvd., Tucson, AZ 85721

**Supplementary Information Text**

**Supplementary Methods.**

***Trait questionnaires.*** The Qualtrics platform was used to provide participants instructions, obtain consent, and initiate the study via an online survey. The survey included questions either developed for the study or included established trait questionnaires. Two questionnaires assessed in the online Qualtrics survey are relevant to the current study: the dream questionnaire (developed by our study team) and the Rumination-Reflection Questionnaire (see Main Manuscript). Participants also completed a number of trait questionnaires within the Mind Window app, only one of which was relevant for the current study: the Ten-Item Personality Inventory.

***Analysis of waking thoughts.*** Ecological Momentary Assessment responses were cleaned and compiled using Jupyter Lab v. 3.0.14 along with the pandas1 and sklearn libraries2. As the data set contains numerous surveys per participant, a single value was obtained for each variable by taking the mean of all available responses for that participant. Some variables were missing responses, with 9 of them having between 98.6% and 99.8% of the intended responses. In order to avoid having to drop participants for having missing responses, Scikit-learn’s Iterative Imputer function was used to estimate scores via multivariate imputation.

***Exploratory factor analysis on participants’ perceived characteristics of their dreams.*** In order to perform the exploratory factor analysis, each variable was first Box-Cox transformed in order to normalize the data as much as possible. Following transformation, each variable fell within acceptable skewness and kurtosis range (|1| and |2|, respectively), with the exception of the intentionality variable which was removed from the analysis due to its multimodal distribution incompatible with the assumption of normality. Additionally, the self-focus and social-orientation variables were removed as a result of low factorability. The remaining variables had adequate individual and overall level of factorability (MSA >.65 and MSA=.69, respectively). The variables were scaled before the exploratory factor analysis was conducted with the *fa* function in the Psych library in R, with the oblimin rotation method. A parallel analysis revealed a 2 factors solution was optimal. This was confirmed by a model comparison of different factor solutions (from 1 to 3) where the 2 factors solution had the lowest BIC (-40.36 vs. 187 for a 1-factor solution and -17.82 for a 3-factor solution).

**Supplementary Results.**

***Statistical comparisons of participants’ ratings of waking thought to those of dreams.*** Paired-samples Wilcoxon-rank-sum tests compared the mean values of each perceived characteristic for each category of waking thought to its dream-related counterpart (**Table S2**). Five perceived characteristics categories for each task-unrelated thought category did not significantly differ with dreams at a Bonferroni corrected alpha of *p* < .001. Specificity, goal-orientation, helpfulness, and valence were all statistically similar to their dream-related counterparts and were statistically equivalent for both stimulus-independent task-unrelated thoughts and stimulus-dependent task-unrelated thoughts. Additionally, stimulus-independent task-unrelated thoughts (but not stimulus-dependent task-unrelated thoughts) persistence and stimulus-dependent task-unrelated thoughts’ (but not stimulus-independent task-unrelated thoughts) awareness were statistically similar to their dream counterpart. On the contrary, the perceived characteristics of both task-related thought categories all significantly differed from dreams after Bonferroni correction except for stimulus-independent task-related thought vividness (*W* = 80288.5, *p* = .034), and persistence (*W* = 98220.5, *p* = .01) and vividness (*W* = 44631.5, *p* = .002) for stimulus-dependent task-related thought. Dreams distinguished themselves as being the highest in self-focus and social orientation, as well as the lowest in intentionality, a difference that was statistically significant when compared to all forms of waking thoughts (all *p*’s <.001). Overall, these analyses confirmed that task-unrelated thought categories – namely stimulus-independent task-unrelated thoughts and stimulus-dependent task-unrelated thoughts – most resembled the profile of dreams.

**Figure S1. Racial & Ethnic background of sample**

**Figure S2. The perceived characteristics of dreams and different categories of waking thought at different cutoff levels.**

Mean ratings for dreams (green solid line) and each of the four waking thought categories. Stimulus dependent thoughts in red and stimulus-independent thought in blue, task-related thought as dotted lines and task-unrelated thought as dashed lines. Across the perceived characteristics, dreams were most similar to stimulus-independent task unrelated thoughts and stimulus-dependent task unrelated thoughts. Note that the anchors for the rating scales were different for some of the characteristics (see Supplementary Table S1). Valence, for example, ranged from 0 (Very Negative) to 1 (Very Positive). Each cutoff represents a requirement to have at least x number of probes in all waking thought categories (e.g., a cutoff of 3 means that only participants who had at least 3 probes in each of stimulus-independent task-unrelated thought, stimulus-dependent task-unrelated thought, stimulus-independent task-related thought and stimulus-dependent task-related thought were included).

**Table S1.** **Questionnaire assessing the phenomenology of participants’ waking thoughts with the Ecological Momentary Assessment app Mind Window**

*Note*: With the exception of vividness, which used a 5 point scale from the Vividness of Visual Imagery Questionnaire, all questions were answered on a sliding scale, converted to a numerical value ranging from 0 to 1. For statistical convenience, the vividness item was divided by 5 to convert it to the same 0 to 1 scale.

**Core Questions**

***stimulus-dependence*** *In the moments just before the notification, where was the focus of your attention?*

- A value from 0 to 1 spread across the input from three interdependent sliders.

_
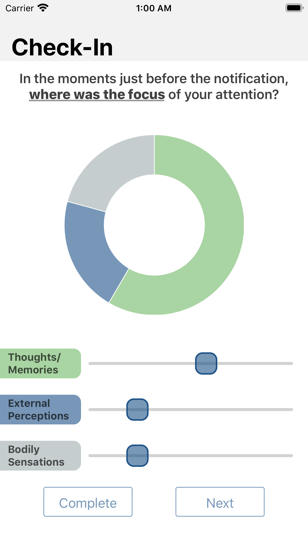
_

*The stimulus-dependence interface within Mind Window.*

***task-relatedness*** *In the moments just before the notification, to what degree were your thoughts/attention focused on your primary “task” or “activity” at hand?*

- Not at All (0)

- A Little

- Somewhat

- Quite a Bit

- Very Much So (1)

***intentionality*** *In the moments just before the notification, to what degree were you actively directing what was on your mind?*

- Not at All (0)

- A Little

- Somewhat

- Quite a Bit

- Very Much So (1)

***awareness*** *In the moments just before the notification, to what degree were you aware of what was on your mind?*

- Not at All (0)

- A Little

- Somewhat

- Quite a Bit

- Very Much So (1)

***persistence*** *Of your thoughts just before the notification, how long do you anticipate that they would have been on your mind? (If the notification had not interrupted you)*

- Just a few seconds (0)

- Several seconds to a minute

- One to several minutes

- Several minutes to an hour

- More than an hour (1)

***temporal specificity*** *In the moments just before the notification, how specific to a particular time were your thoughts?*

- No Time (0)

- General or Vague Time

- Very Specific Time (1)

***spatial specificity*** *In the moments just before the notification, how specific to a particular place were your thoughts?*

- No Place (0)

- General or Vague Place

- Very Specific Place (1)

***valence*** *In the moments just before the notification, how positive or negative were your thoughts?*

- Very negative (0)

- Somewhat negative

- Neutral

- Somewhat positive

- Very positive (1)

***self-focus,***  *In the moments just before the notification, to what degree were your* ***social orientation*** *thoughts about you? To what degree were they about other people?*

- A visual interface where users could indicate different levels of self or other thinking. An empty figure was converted to 0 while a full-figure was converted to 1 (on a continuous scale).


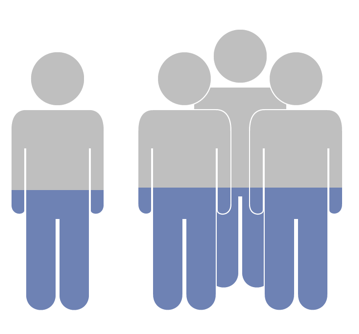


*The self-focus and social-orientation interface within Mind Window*

**Rotating Questions**

***vividness*** *In the moments before the notification, overall, how vivid were your thoughts in your "mind's eye?"*

- No image at all (you only "know" you are thinking of something) (0)

- Vague and dim

- Moderately clear and vivid

- Clear and reasonably vivid

- Perfectly clear and vivid as normal vision (5)

***goal-orientation*** *In the moments just before the notification, to what degree were your thoughts oriented to a goal, or multiple goals, of yours?*

- Not at All (0)

- A Little

- Somewhat

- Quite a Bit

- Very Much So (1)

***helpfulness*** *Regarding your thoughts in the moments just before the notification, how productive or helpful do you think they were?*

- Very Unproductive (0)

- Mostly Unproductive

- Hard to Tell

- Productive

- Very Productive (1)

**Table S2.** **Dream questionnaire assessing the phenomenology of participants’ typical dreams**

***Instructions***: “*We are interested in the kinds of dreams you usually have at night. Please answer the following questions to the best of your ability about the* ***typical*** *nature of the dreams that you remember.”*

*Note*: With the exception of vividness, which used a 5-point scale from the Vividness of Visual Imagery Questionnaire, all questions were answered on a sliding scale from 0 to 1 with the anchors for each question shown below each question. For statistical convenience, the vividness item was divided by 5 to convert it to the same 0 to 1 scale.

***intentionality*** *To what degree are you able to consciously direct the focus, or content, of your dreams?*

- Not at All (0)

- A Little

- Somewhat

- Quite a Bit

- Very Much So (1)

***awareness*** *To what degree are you aware that you are dreaming, even as you dream?*

- Not at All (0)

- A Little

- Somewhat

- Quite a Bit

- Very Much So (1)

***persistence*** *How long do your dreams usually stay on your mind?*

- Just a few seconds (0)

- Several seconds to a minute

- One to several minutes

- Several minutes to an hour

- More than an hour (1)

***vividness*** *How vivid are your dreams in your "mind's eye?” (single choice Likert-type)*

- No image at all (you only "know" you are thinking of something) (1)

- Vague and dim

- Moderately clear and vivid

- Clear and reasonably vivid

- Perfectly clear and vivid as normal vision (5)

***temporal***  *How specific to a particular time are your dreams?*

***specificity***

- No Time (0)

- General or Vague Time

- Very Specific Time (1)

***spatial*** *How specific to a particular place are your dreams?*

***specificity***

- No Place (0)

- General or Vague Place

- Very Specific Place (1)

***goal-*** *To what degree are your dreams oriented towards your goals?*

***orientation***

- Not at All (0)

- A Little

- Somewhat

- Quite a Bit

- Very Much So (1)

***helpfulness*** *How productive or helpful are your dreams?*

- Very Unproductive (0)

- Mostly Unproductive

- Hard to Tell

- Productive

- Very Productive (1)

***valence*** *How positive or negative are your dreams?*

- Very negative (0)

- Somewhat negative

- Neutral

- Somewhat positive

- Very positive (1)

***self-focus*** *To what degree are your dreams about you?*

- None (0)

- Half

- All (1)

***social-orientation*** *To what degree are your dreams about other people?*

- None (0)

- Half

- All (1)

**Table S3. Mean comparisons between dreams and different categories of waking thoughts.**

Wilcoxon-rank-sum tests were computed to compare the perceived characteristics of dreams to each of 4 categories of waking thoughts. Waking thoughts characterized by their task-unrelatedness (SITUT and SDTUT) were most similar to dreams. The mean comparisons were replicated at different cutoff, each representing a requirement to have at least 1, 2, 3, or 4 probes in all waking thought categories (e.g., a cutoff of 3 means that only participants who had at least 3 probes in each of SITUT, SITRT, SDTUT, and SDTRT were included). SITUT = stimulus-independent task-unrelated thoughts; SDTUT = stimulus-dependent task-unrelated thoughts; SITRT = stimulus-independent task-related thoughts; SDTRT = stimulus-dependent task-related thoughts; *p <.05, **p<.01, ***p <.001 (Bonferroni corrected p-value threshold)

|  |  | Thought Characteristic | | | | | | | | | | |
| --- | --- | --- | --- | --- | --- | --- | --- | --- | --- | --- | --- | --- |
|  | Dreams vs. | **Intentionality** | **Social-Orientation** | **Self-Focus** | **Vividness** | **Persistence** | **Valence** | **Helpfulness** | **Awareness** | **Goal-Orientation** | **Specificity** | **Awareness** |
| No Cutoff  (N = 719) | SITUT | 81,752*** | 162,023*** | 164,104.5*** | 5,9856*** | 132,842** | 118,784 | 58,504 | 42,637*** | 61,649.5 | 100,743.5** | 42,637*** |
|  | SDTUT | 50,531*** | 83,944.5*** | 100,528.5*** | 16,130.5*** | 82,102.5*** | 55,973.5** | 18,348 | 15,226* | 18,499.5** | 67,945 | 15,226* |
|  | SITRT | 24,804.5*** | 177,258*** | 165,243.5*** | 81,288.5* | 99,007*** | 68121.5*** | 25,472.5*** | 22,820*** | 37,411*** | 70,439*** | 22,820*** |
|  | SDTRT | 21,246*** | 145,989.5*** | 150,169*** | 44,631** | 98,220.5 | 49268*** | 13,599*** | 17,634*** | 24,112*** | 72,286*** | 17,634*** |
| Cutoff = 1  (N = 482) | SITUT | 40,039*** | 78,819*** | 81,480.5*** | 33,212.5*** | 65,933** | 57,509 | 32,088.5 | 23,495.5*** | 33,689.5 | 51,038.5* | 23,495.5*** |
|  | SDTUT | 45,444.5*** | 72,547.5*** | 89,751.5*** | 14,234.5*** | 72,659.5*** | 48269** | 16,845 | 14,096 | 16,360* | 58,993.0 | 14,096 |
|  | SITRT | 11,170.5*** | 80,373*** | 76,483.5*** | 37,170.5*** | 46,631*** | 32309.5*** | 11,839.5*** | 9,553*** | 17,940*** | 32,580.5*** | 9,553*** |
|  | SDTRT | 12,176.5*** | 81,000.5*** | 82,357.5*** | 29,526.5*** | 54,968 | 28725*** | 8,779*** | 11,480*** | 14,977.5*** | 41,076*** | 11,480*** |
| Cutoff = 2  (N = 327) | SITUT | 19,192*** | 37,565*** | 37,920*** | 16,021.5*** | 30,935.5** | 24,308.0 | 16,256.5 | 12,301.5*** | 16,416.0 | 24,743.5 | 12,301.5*** |
|  | SDTUT | 21,597.5** | 35,028.5*** | 41,422*** | 10,066*** | 34,574*** | 20,129*** | 12,194* | 9,844 | 10,728.5 | 28,898 | 9,844 |
|  | SITRT | 52,38.5*** | 38,419.5*** | 34,403.5*** | 17,106** | 21,246.5** | 13,640.5*** | 5,624.5*** | 4,606.5*** | 8,262.5*** | 16,639*** | 4,606.5*** |
|  | SDTRT | 5,537*** | 37,934*** | 37,841.5*** | 16,017.5** | 24,570 | 11,166*** | 5,331.5*** | 6,450*** | 7,595.5*** | 19,904*** | 6,450*** |
| Cutoff = 3  (N = 216) | SITUT | 8,391.5*** | 16,560*** | 16,792.5*** | 7,172.5*** | 13,777* | 10,958 | 6,898 | 5,848** | 7,358.0 | 11,300 | 5,848** |
|  | SDTUT | 9,305.5* | 16,089.5*** | 18,419*** | 5,917*** | 15,556.5*** | 8,292*** | 6,223.5* | 6,280.5 | 5,921.5 | 13,427 | 6,280.5 |
|  | SITRT | 2,380.5*** | 17,289.5*** | 15,402*** | 7,477* | 9,091.5** | 6,575*** | 2,225*** | 2,396*** | 3,747.5*** | 8,270.5*** | 2,396*** |
|  | SDTRT | 2,254*** | 17,246.5*** | 16,627*** | 7,670.5* | 10,769 | 5,235.5*** | 2,578*** | 3,422*** | 4,010*** | 10,139 | 3,422*** |
| Cutoff = 4  (N = 142) | SITUT | 3,968* | 6,932.5*** | 7,206*** | 3,196.5*** | 6,074.5* | 5,153.5 | 3,270.5 | 2,994.0 | 3,718.5* | 4,598 | 2,994.0 |
|  | SDTUT | 4,473.0 | 6,922*** | 7,983*** | 3,109.5*** | 6,704.5*** | 3,861.5* | 3,004.0 | 3,398.5 | 3,346* | 5,635 | 3,398.5 |
|  | SITRT | 1,206*** | 7,411.5*** | 6,795*** | 3,482* | 3,783** | 3,068.5*** | 966.5*** | 1,012*** | 1,586.5*** | 3,492** | 1,012*** |
|  | SDTRT | 1,249*** | 7,376*** | 7,365.5*** | 3,950* | 4,461 | 2,538*** | 1,036.5*** | 1,523.5*** | 1,926.5*** | 4,116 | 1,523.5*** |

**Table S4. Spearman Rank correlations between task-unrelated thought and dream perceived characteristics.**

Replication of Table 1 with a cutoff of at least 4 task-unrelated thought probes for each participant.

| **Characteristic** | **Spearman's *ρ*** | ***p-*value** | **n** |
| --- | --- | --- | --- |
| Intentionality | 0.21 | **<0.0001** | 544 |
| Social | 0.16 | **0.0003** | 544 |
| Self-Focus | 0.05 | 0.215 | 544 |
| Vividness | 0.08 | 0.0823 | 487 |
| Persistence | 0.15 | **0.0003** | 544 |
| Valence | 0.26 | **<0.0001** | 544 |
| Helpfulness | 0.25 | **<0.0001** | 500 |
| Goal-Orientation | 0.13 | **0.0041** | 486 |
| Specificity | 0.07 | 0.0966 | 544 |
| Awareness | 0.07 | 0.1138 | 490 |
| Bonferroni Correction Factor = 0.005 | |  |  |

**Table S5.** **Relationships between COVID-19 concern scores and dream perceived characteristics (n = 429).**

| **Characteristic** | ***r*** | ***p*** |
| --- | --- | --- |
|  |  |  |
| Dream Intentionality | -0.086 | 0.075 |
| Dream Social Orientation | 0.014 | 0.76 |
| Dream Self-Focus | -0.042 | 0.38 |
| Dream Vividness | -0.050 | 0.31 |
| Dream Persistence | 0.001 | 0.98 |
| Dream Valence | -0.181 | **<0.001** |
| Dream Helpfulness | -0.145 | **0.003** |
| Dream Goal-Orientation | -0.143 | **0.003** |
| Dream Specificity | -0.013 | 0.79 |
| Dream Awareness | -0.073 | 0.13 |

**Table S6.** **Relationships between trait rumination scores and dream perceived characteristics (n = 719).**

| **Characteristic** | ***r*** | ***p*** |
| --- | --- | --- |
|  |  |  |
| Dream Intentionality | -0.014 | 0.72 |
| Dream Social Orientation | 0.028 | 0.45 |
| Dream Self-Focus | -0.010 | 0.79 |
| Dream Vividness | 0.040 | 0.28 |
| Dream Persistence | 0.153 | **<0.001** |
| Dream Valence | -0.274 | **<0.001** |
| Dream Helpfulness | -0.128 | **0.001** |
| Dream Goal-Orientation | -0.177 | **<0.001** |
| Dream Specificity | -0.001 | 0.98 |
| Dream Awareness | -0.069 | 0.063 |

**Table S7. Relationships between personality scores and dream perceived characteristics (n = 698).**

Neuroticism, extraversion, conscientiousness, agreeableness and openness to experience scores from the Ten-Item Personality Inventory were examined in relation to each of two dream factor scores.

|  | **Negative Unconstructive Dream Factor Score** | | **Immersive Dream**  **Factor Score** | |
| --- | --- | --- | --- | --- |
|  | ***r*** | ***p*** | ***r*** | ***p*** |
| **Neuroticism** | 0.21 | **<0.001** | -0.12 | **0.001** |
| **Extraversion** | -0.14 | **<0.001** | -0.04 | 0.269 |
| **Conscientious** | -0.11 | **0.003** | 0.02 | 0.517 |
| **Agreeable** | -0.03 | 0.377 | -0.01 | 0.762 |
| **Openness** | 0.15 | **<0.001** | -0.12 | **0.001** |

**Table S8. Controlling for the effect of personality scores on dreams.**

Neuroticism, extraversion, conscientiousness, agreeableness and openness to experience scores from the Ten-Item Personality Inventory were included as covariates in 12 separate models predicting task-unrelated thoughts (TUT), Trait Rumination and COVID-19 concern from relevant dream characteristics. For simplicity, results are only shown for the dream predictors. With the exception of COVID-19 concern, which became a marginal relationship when controlling for personality, associations between dreams and outcomes that were previously significant remained significant, and those that were previously not significant remained not significant.

| **Model #** | **Outcome** | **Main Predictor** | **β** | **Error** | ***t*** | ***p*** |
| --- | --- | --- | --- | --- | --- | --- |
|  |  |  |  |  |  |  |
| 1* | TUT Intentionality | Dream Intentionality | 0.095 | 0.022 | 4.347 | <0.001 |
| 2* | TUT Social Orientation | Dream Social Orientation | 0.116 | 0.028 | 4.132 | <0.001 |
| 3 | TUT Self-Focus | Dream Self-Focus | 0.025 | 0.031 | 0.799 | 0.424 |
| 4 | TUT Vividness | Dream Vividness | 0.046 | 0.047 | 0.986 | 0.325 |
| 5* | TUT Persistence | Dream Persistence | 0.074 | 0.022 | 3.42 | <0.001 |
| 6* | TUT Valence | Dream Valence | 0.112 | 0.025 | 4.462 | <0.001 |
| 7* | TUT Helpfulness | Dream Helpfulness | 0.24 | 0.04 | 5.964 | <0.001 |
| 8* | TUT Goal-Orientation | Dream Goal-Orientation | 0.129 | 0.045 | 2.852 | 0.005 |
| 9* | TUT Specificity | Dream Specificity | 0.071 | 0.032 | 2.192 | 0.029 |
| 10 | TUT Awareness | Dream Awareness | 0.046 | 0.037 | 1.229 | 0.219 |
|  |  |  |  |  |  |  |
| 11* | Trait Rumination | Negative Unconstructive Dream Factor Score | 0.119 | 0.032 | 3.755 | <0.001 |
|  |  | Immersive Dream Factor Score | 0.114 | 0.033 | 3.515 | <0.001 |
|  |  |  |  |  |  |  |
| 12* | COVID-19 Concern | Negative Unconstructive Dream Factor Score | 0.021 | 0.011 | 1.921 | 0.055 |

* = The model was significant at *p*<.05 when not controlling for personality factors (see Table 1 from the main manuscript).
